# Supplementary material for: Design and Synthesis of a Cyclic Double-Grafted Polymer Using Active Ester Chemistry and Click Chemistry via A “Grafting onto” Method
Source: Polymers (Basel). 2019 Feb 1;11(2):240. doi: 10.3390/polym11020240 (PMC6419024; doi:10.3390/polym11020240)
Supplement: Supplementary file 1 [file polymers-11-00240-s001.pdf]

Supporting Information for

Design and Synthesis of a Cyclic Double-Grafted Polymer Using Active Ester Chemistry and Click Chemistry via a "Grafting onto" Method

Meng Liu [1], Lu Yin [1], Shuangshuang Zhang [1,*], Zhengbiao Zhang [1], Wei Zhang [1,*] and Xiulin Zhu [2]

Experiment part

1.1 Synthesis routes

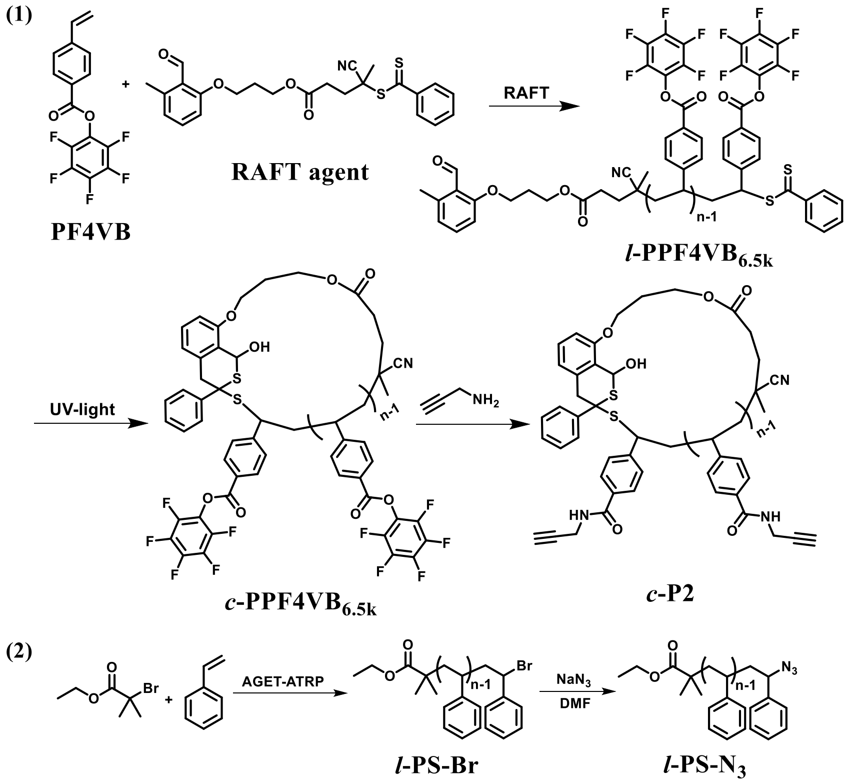

Scheme S1. Illustration of synthetic routes of cyclic polymer (*c*-PPF4VB$_{6.5k}$) and linear polystyrene (*l*-PS-N_3).

1.2 Synthesis of RAFT agent, monomer (PF4VB) and azide-functional polystyrene (*l*-PS-N_3)

Functional RAFT agent, monomer (pentafluorophenyl 4-vinylbenzoate, PF4VB) and azide-functional polystyrene (*l*-PS-N_3) were synthesized and characterized according to our previous publication [1].

1.3 Synthesis of linear poly(pentafluorophenyl 4-vinylbenzoate) (*l*-$PPF4VB_{6.5k}$) [1]

Monomer (PF4VB) (6.28 g, 20 mmol) and RAFT-agent (91.1 mg, 0.2 mmol) were dissolved in 10 mL toluene. The mixture was degassed by freeze-thaw cycling and then placed into an oil bath at 110 °C for 7.5 h. The polymerization was stopped by cooling and poured into 200 mL of hexane. The polymer was gained by filtration and dried under vacuum at 30 °C for 24 h (1.47 g, conv.%: 23.0%, $M_{n,th}$ = 7700 g/mol, $M_{n,SEC}$ = 6500 g/mol, M_w/M_n = 1.05, $M_{n,NMR}$ = 8900 g/mol).

1.4 Synthesis of cyclic poly(pentafluorophenyl 4-vinylbenzoate) (*c*-$PPF4VB_{6.5k}$) [1]

The polymer (l-$PPF4VB_{6.5k}$) (15 mg) and mixture solvent of DCM (100 mL)/acetonitrile 300 (mL) were added into a round-bottom flask under nitrogen atmosphere. The solution was irradiated for 12 h under UV-light (120 W, CEL-LPH120-254, Beijing China Education Au-light co. Ltd) at ambient temperature. The solution was evaporated and precipitated in cold hexane. The polymer was collected by filtration and dried under vacuum. (12.0 mg, yield: 80%, $M_{n,SEC}$ = 6200 g/mol, M_w/M_n = 1.06). The crude cyclic polymer was further purified by preparative SEC.

1.5 Synthesis of cyclic poly(*N*-(prop-3-ynyl)-exo-4-vinylbenzamide) (*c*-P2)

The polymer (*c*-$PPF4VB_{6.5k}$) (45 mg, 5×10^{-3} mmol), propargylamine (29.7 mg, 0.54 mmol) and THF (600 μL) were added into a 2 mL ampoule with a magnetic stirrer under nitrogen atmosphere. The solution was stirred at room temperature for 12 h. The polymer was precipitated in hexane three times. The post-modified polymer (*c*-P2) was collected by filtration and dried under vacuum (24.2 mg, $M_{n,SEC}$ = 4400 g/mol, M_w/M_n = 1.08).

Characterization

The number average molecular weights (M_n) and molecular weight distribution (M_w/M_n) of all the polymers were measured using TOSOH HLC-8320 size exclusion chromatography (SEC) equipped with a refractive-index detector, using two TSKgel SuperMultiporeHZ-N (4.6 × 150 mm, 3.0 μm beads size) columns with molecular weight ranging from 500 to 1.9×10^5 Da. Tetrahydrofuran (THF) was used as eluent at a flow rate of 0.35 mL/min at 40 °C. Polystyrenes were used as standard samples. For crude polymers, recycling preparative HPLC Mode LC-9260NEXT (often called as Prep-SEC) equipped with a manual injector, UV-vis 4ch (200–800 nm) and RI-700NEXT detectors, using JAIGEL-2HH and JAIGEL-2.5HH (PS/DVB packing material) columns, was utilized to purify samples. THF was used as eluent with a flow rate of 6 mL/min at room temperature. All the ^{1}H NMR and ^{13}C NMR spectra were recorded on a Bruker Nuclear Magnetic Resonance instrument (300 MHz) with tetramehylsilane (TMS) as internal

standard and $CDCl_3$ as solvent at 25 °C. Matrix assisted laser desorption ionization/time of flight (MALDI TOF) mass spectra were acquired on an UltrafleXtreme MALDI TOF mass spectrometer equipped with a 1 kHz smart beam-II laser. The compound *trans*-2-[3-(4-*tert*-butyl-phenyl)-2-methyl-2-propenylidene]-malononitrile (DCTB, Aldrich, >98%) in $CHCl_3$ (20 mg/mL) was used as matrix. Sodium trifluoroacetate was considered as cationizing agent (20 mg/mL in ethanol). All samples were dissolved in $CHCl_3$ as a concentration of 10 mg/mL.

Discussion

For linear polymer (*l*-PPF4VB$_{6.5k}$), ^{1}H NMR spectroscopy (Figure S2) shows typical signals from RAFT agent at 10.62 (-*ArCHO*, a), 7.26-7.60 (-*PhH*, h), 4.27 (*ArOCH$_2$*-, e), 4.1 (-*COOCH$_2$*-, g) and 2.54 ppm (-*ArCH$_3$*, b). The corresponding a/h/e/g/b integration ratio is close to 1/5/2/2/3, confirming the fidelity of thiocarbonyl benzene groups and orthoquinodimethane. In addition, the observed signals from ^{13}C NMR spectroscopy (Figure S3) further indicated the fidelity of the end groups. The number average molecular weight ($M_{n,NMR}$ = 8900 g/mol, Figure S2) are calculated by comparing integrated signals at 4.22 and 7.93 ppm. SEC curve (Figure S4) exhibited a monomodal and symmetric peak. The molecular weight of $M_{n,SEC}$ (6500 g/mol) was directly calculated by polystyrene standards without using the Mark-Houwink parameters. MALDI TOF MS (Figure S5) exhibited three main peak distributions. These main peak distributions were accurately assigned to linear polymer (*l*-PPF4VB$_{6.5k}$) having different chain ends. The representative experimental *m/z* value (such as 5817.11 Da) from 17 repeating units of polymer with one sodium cation, is consistent with theoretically calculated value ($[17M+Na]^+$, 5816.73 Da). The mass difference between two adjacent peaks (313.07 Da) accords with the value of one repeating unit. These results demonstrated the successful preparation of linear polymer (*l*-PPF4VB$_{6.5k}$).

Under UV-light resource, linear polymer (*l*-PPF4VB$_{6.5k}$) was used for preparation of cyclic polymer (*c*-PPF4VB$_{6.5k}$) in highly diluted solution. ^{1}H NMR spectroscopy (Figure S2) showed that the characteristic signal corresponding to -*ArCHO* (10.62 ppm, a) disappeared and a new peak was observed at 5.34 ppm, assigned to the methine hydrogen (a′) adjacent to the hydroxyl group after cyclization. In comparison with their linear counterparts (*l*-PPF4VB$_{6.5k}$) (Figure S4), the SEC curve of cyclic polymer (*c*-PPF4VB$_{6.5k}$) shifted to low molecular weight region completely due to its smaller hydrodynamic volume. Additionally, the π-π* adsorption peak of thiocarbonyl moiety of RAFT agent and *l*-PPF4VB$_{6.5k}$ in UV-vis spectroscopy (Figure S6) disappeared completely after cyclization. The color of polymer also changed from pink to white after cyclization (Figure S7). Combining all the above characterization data, the successful preparation of cyclic polymer (*c*-PPF4VB$_{4.0k}$) can be verified.

By virtue of reacting cyclic polymer (*c*-PPF4VB$_{6.5k}$) with propargylamine,

functionalized polymer (*c*-P2) could be easily obtained without any purification. Figure S8 shows the ^{1}H NMR spectrum of *c*-P2. The new characteristic peaks from propargyl group and amide group (c, d, e) were clearly observed. The integration ratio of a (-*ArH*), b (-*ArH*), c (-*CONH*-), d (-CH_2-) and e (-*CCH*) was approximately 2/2/1/2/1, demonstrating the quantitative reaction conversion. SEC curves (Figure S4) exhibited that *c*-P2 shifted to smaller molecular weight region compared to *c*-$PPF4VB_{6.5k}$, while maintaining narrow molecular weight distribution (M_w/M_n = 1.08).

Reference

1. Zhang, S.; Cheng, X.; Wang, J.; Zhang, Z.; Zhang, W.; Zhu, X. Synthesis of a cyclic-brush polymer with a high grafting density using active ester chemistry via the "grafting onto" approach. *Polym. Chem.* **2018**, *9*, 5155-5163.

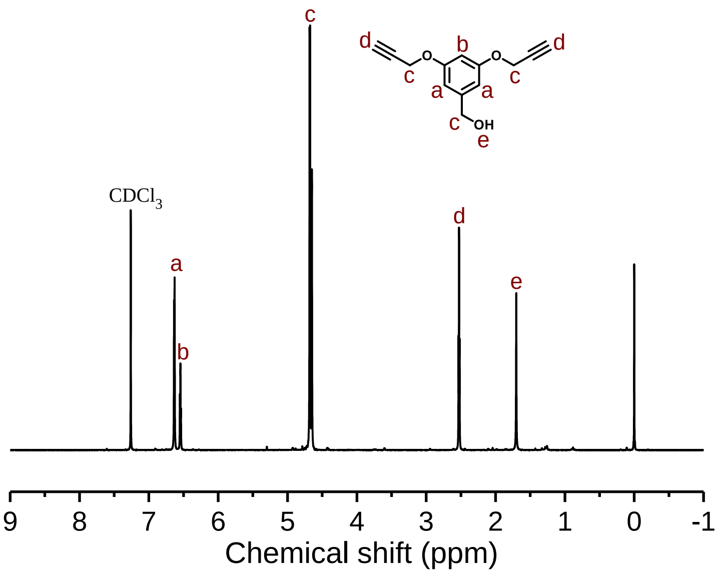

Figure S1. ^{1}H NMR (300 MHz) spectrum of 3,5-bis(propargyloxy)benzyl alcohol.

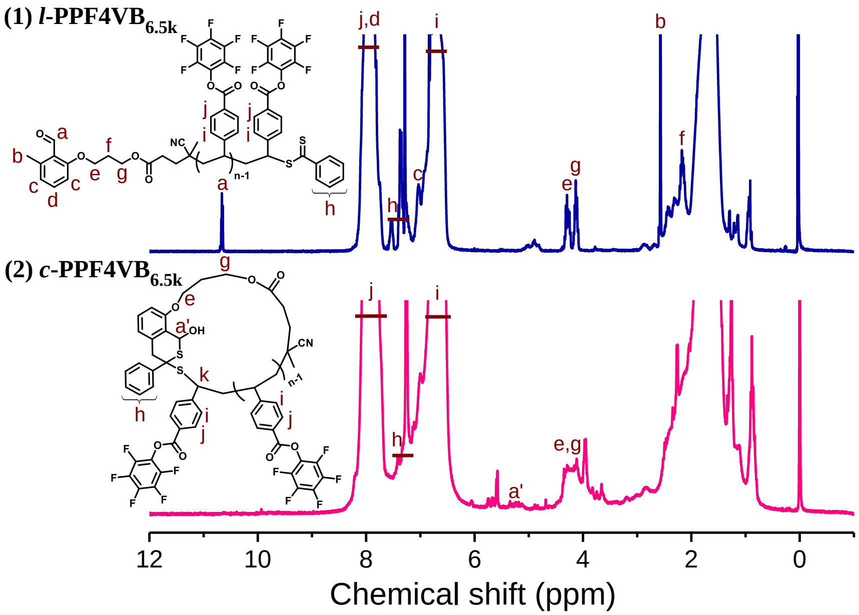

Figure S2. ^{1}H NMR (300 MHz) spectra of linear polymer (*l*-PPF4VB$_{6.5k}$) and cyclic polymer (*c*-PPF4VB$_{6.5k}$).

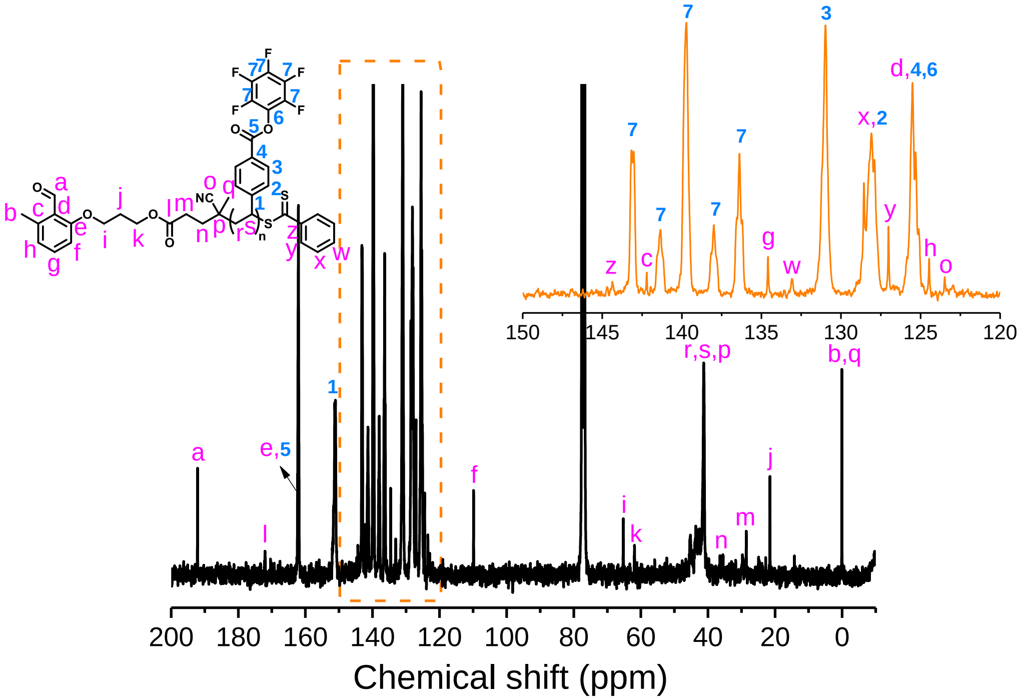

Figure S3. ^{13}C NMR (75 MHz) spectrum of linear polymer (*l*-PPF4VB$_{4.0k}$).

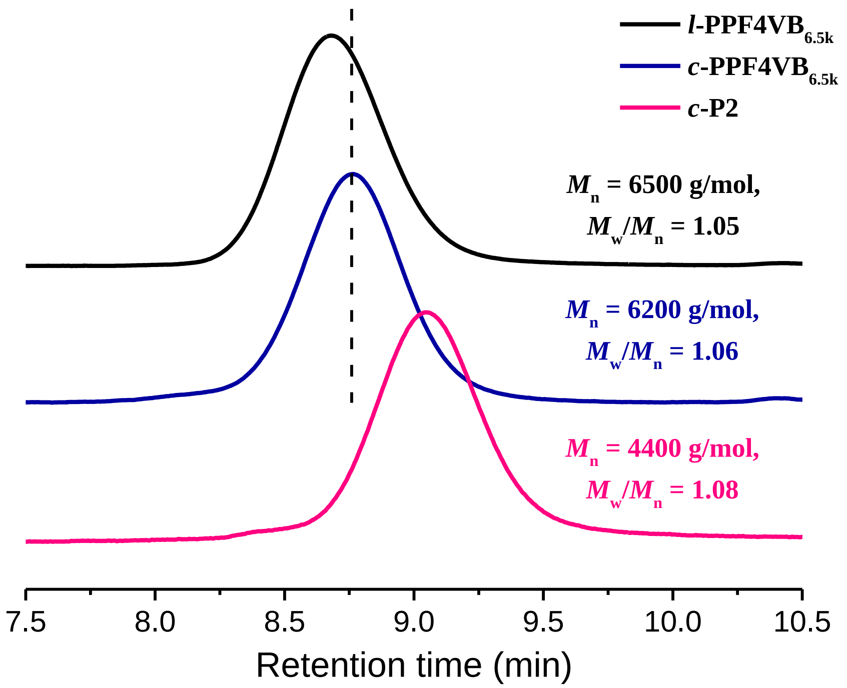

Figure S4. SEC curves of *l*-PPF4VB4.0k, *c*-PPF4VB4.0k and *c*-P2. THF was used as the eluent and PS standards for the calibration.

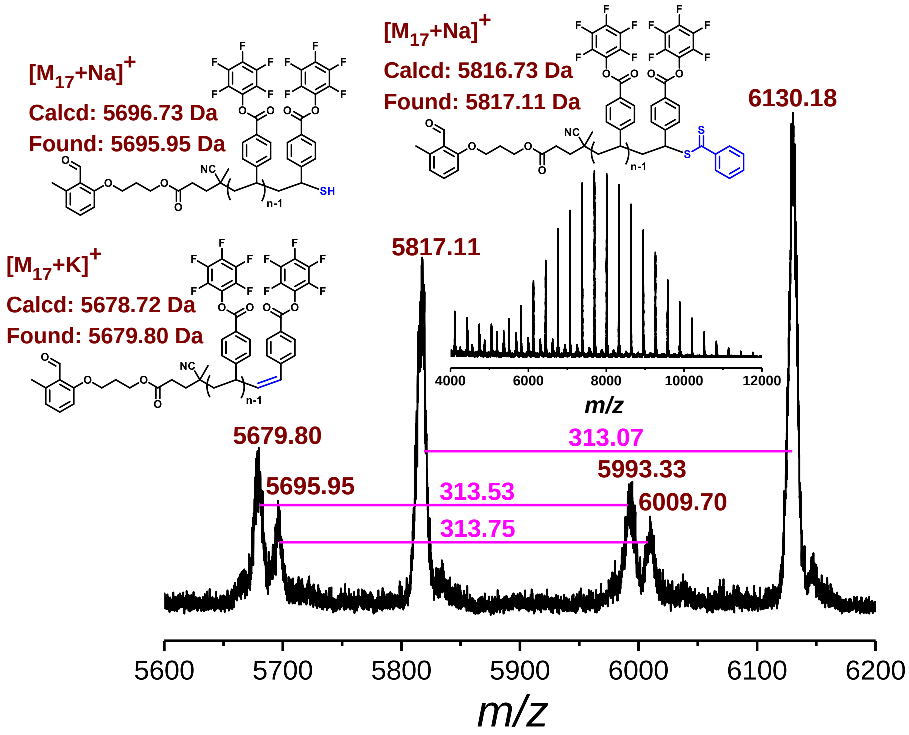

Figure S5. Expanded MALDI-TOF mass spectrum of *l*-PPF4VB6.5k with inserted full spectrum.

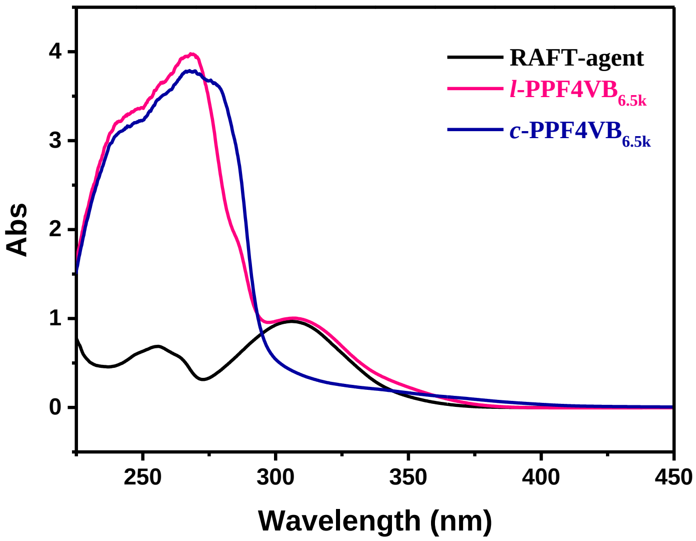

Figure S6. UV-vis spectrum of RAFT agent, *l*-PPF4VB$_{6.5k}$ and *c*-PPF4VB$_{6.5k}$.

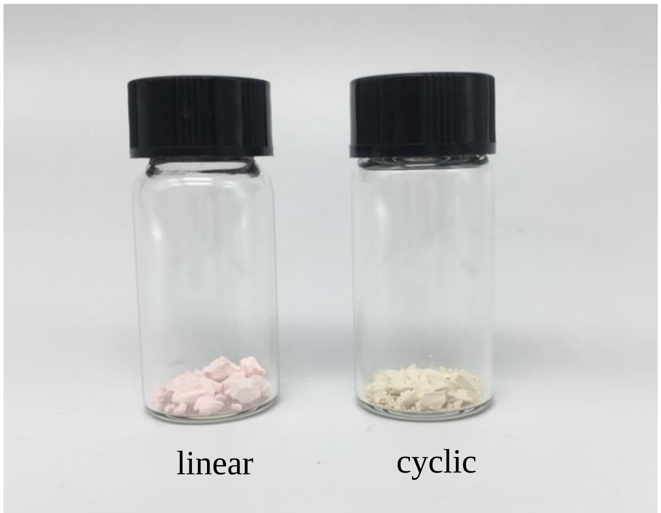

Figure S7. Pictures of *l*-PPF4VB$_{6.5k}$ and *c*-PPF4VB$_{6.5k}$.

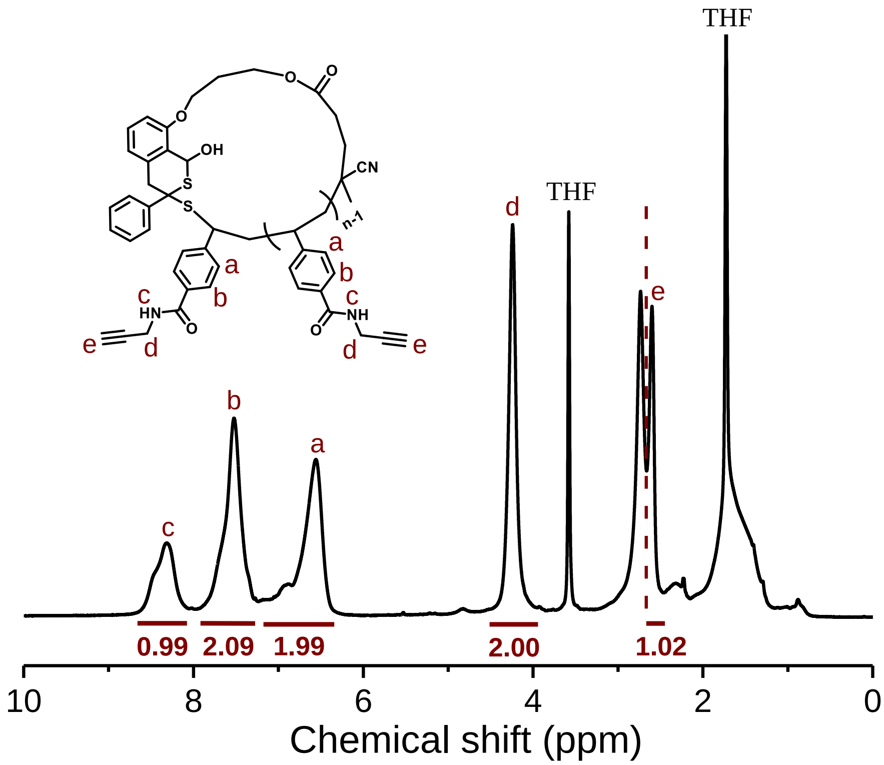

Figure S8. ^{1}H NMR (300 MHz) spectrum of cyclic polymer (*c*-P2).
